# A Mad7 System for Genetic Engineering of Filamentous Fungi

**DOI:** 10.3390/jof9010016

**Published:** 2022-12-22

**Authors:** Katherina Garcia Vanegas, Jakob Kræmmer Haar Rendsvig, Zofia Dorota Jarczynska, Marcio Vinicius de Carvalho Barros Cortes, Abel Peter van Esch, Martí Morera-Gómez, Fabiano Jares Contesini, Uffe Hasbro Mortensen

**Affiliations:** 1 Eukaryotic Molecular Cell Biology, Section for Synthetic Biology, Department of Biotechnology and Biomedicine, Technical University of Denmark, Søltofts Plads, 2800 Kongens Lyngby, Denmark; 2 Embrapa Rice and Beans–Brazilian Agricultural Research Corporation, Santo Antônio de Goiás 75375-000, GO, Brazil

**Keywords:** *Aspergillus*, CRISPR, Mad7, fungal strain engineering

## Abstract

The introduction of CRISPR technologies has revolutionized strain engineering in filamentous fungi. However, its use in commercial applications has been hampered by concerns over intellectual property (IP) ownership, and there is a need for implementing Cas nucleases that are not limited by complex IP constraints. One promising candidate in this context is the Mad7 enzyme, and we here present a versatile Mad7-CRISPR vector-set that can be efficiently used for the genetic engineering of four different *Aspergillus* species: *Aspergillus nidulans, A. niger*, *A. oryzae* and *A. campestris*, the latter being a species that has never previously been genetically engineered. We successfully used Mad7 to introduce unspecific as well as specific template-directed mutations including gene disruptions, gene insertions and gene deletions. Moreover, we demonstrate that both single-stranded oligonucleotides and PCR fragments equipped with short and long targeting sequences can be used for efficient marker-free gene editing. Importantly, our CRISPR/Mad7 system was functional in both non-homologous end-joining (NHEJ) proficient and deficient strains. Therefore, the newly implemented CRISPR/Mad7 was efficient to promote gene deletions and integrations using different types of DNA repair in four different *Aspergillus* species, resulting in the expansion of CRISPR toolboxes in fungal cell factories.

## 1. Introduction

Filamentous fungi are extensively used as producers of industrial enzymes and metabolites that are applied by the food and pharma industries [1,2]. In this context, it is important to stress that genome sequencing studies have demonstrated that the potential of new product discovery in fungi is vast [3,4,5]. A bottleneck in exploiting the full fungal production potential is the lack of genetic engineering tools that enable efficient product discovery and process optimization. The recent introduction of CRISPR technology in fungi promises to reduce this barrier, as efficient gene-editing methods based on CRISPR nucleases such as Cas9 and Cpf1 (Cas12a) have demonstrated their widespread functionality and applications in many different fungal species [6,7,8,9]. CRISPR-based gene editing can be achieved by exploiting that programmed CRISPR nuclease-induced DNA double-strand breaks (DNA DSBs) are mostly repaired by pathways based on non-homologous end-joining (NHEJ) or homologous recombination (HR) [10,11,12,13]. Introduction of unspecific mutations at a defined genomic site can be achieved when CRISPR nuclease-induced DNA DSBs are repaired by flawed NHEJ repair. In this case, unspecific mutation of the target site typically disrupts a cycle of accurate DNA DSB repair by NHEJ or HR followed by re-cutting of the restored target site by the CRISPR nuclease. Hence, implementation of the mutation typically takes some generations [14]. Introduction of specific mutations require that the CRISPR nuclease-induced DNA DSB are repaired by HR using a tailor-made repair template, RT. Typically, RTs are classical gene-targeting substrates, but other substrates such as single-stranded oligonucleotides can also be used [15]. The success rate of this type of gene editing is reflecting competition between repairing the CRISPR nuclease-induced DNA DSB by HR using the desired RT or by flawed NHEJ. Hence, highly efficient specific CRISPR-based mutagenesis can be achieved by eliminating the NHEJ pathway in the target species [15]. A well-known mutation that results in NHEJ-deficient strains is the deletion of the *ku70* gene [16].

**Figure 1:**
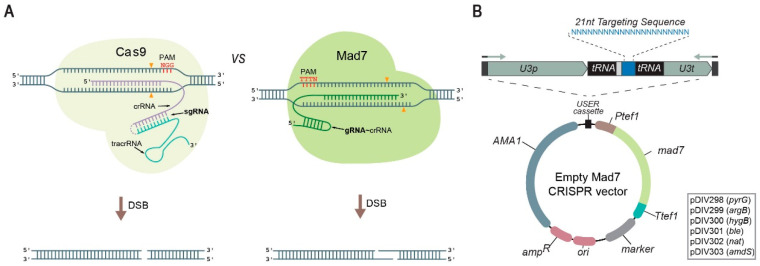
A Mad7 system for genetic engineering of fungi. (**A**) Schematic representations of Cas9 (left) and Mad7 (right) CRISPR nucleases. Scissile bonds in the target sequences are marked with orange triangles [24,25]. The positions of PAM sequences, as well as the orientation and the pairing of the sgRNA of Cas9 (fusion of tracrRNA and crRNA is indicated by the dotted line) and the gRNA of Mad7 (note that Mad7 has a natural single gRNA(=crRNA)) to the target template, are shown. (**B**) Mad7 vectors available for fungal genetic engineering. The vectors are shuttle vectors that can propagate in *E. coli* using ampicillin selection and in fungi using the AMA1 sequence and one of the selection markers indicated in the box. All vectors contain a *mad7* gene and a USER cassette for insertion of the gRNA encoding gene by e.g., USER fusion of two PCR fragments, see Figure S1. Expression of the gRNA gene results in a composite transcript where the gRNA is released by the endogenous tRNA maturation machinery; see text for details.

Despite the rapid implementation of CRISPR technology in a broad range of fungi [14,17,18], uncertainties in IP ownership, royalty complications and license fees constrain the full use of CRISPR technology in the industry [7,19]. Hence, it is desirable to expand the repertoire of efficient CRISPR nucleases with no or less IP issues. One alternative CRISPR nuclease, Mad7 (also known as ErCas12a), is accompanied by a straightforward license that allows free use for commercial and academic research and for development purposes [19]. Mad7 is a CRISPR nuclease based on a codon-optimized gene from the *Eubacterium rectale* (refseq WP_055225123.1). The codon-optimized *mad7* gene shows 76% identity to the native *E. rectale* nucleotide sequence and encodes for a monomeric protein composed by 1263 amino acid residues with a molecular weight of 147.9 kDa. Similar to Cpf1 but not Cas9, Mad7 naturally employs a single RNA species to guide it to the target DNA sequence and it creates DNA DSB with sticky ends rather than blunt ends. Mad7 displays a preference for a 5′-TTTN-3′ PAM site [20] rather than 5′-NGG-3′, which is preferred by Cas9, see Figure 1A. We have previously demonstrated that Mad7 can be used to introduce a gene-expression cassette into a synthetic targeting site present in a set of *Aspergillus* strains [21]. However, no versatile system has so far been developed for use of Mad7 in filamentous fungi. Here, we present six different AMA1-based Mad7-CRISPR vectors that contain different selectable markers, and which can easily be reprogrammed to encode new gRNA species (Figure 1B) via USER cloning or other methods including Gibson- or in vivo assembly [22,23]. Moreover, we show that Mad7 can be used to support a range of gene-editing reactions in four different Aspergilli including a wild-type isolate from a species, which has not previously been genetically engineered.

## 2. Materials and Methods

### 2.1. Strains and Media

Propagation of all plasmids was performed in *Escherichia coli* strain DH5α. The cells were cultured for 12 h at 37 °C on Luria broth (LB) plates, prepared with 25 g/L of LB with agar and supplemented with 100 μg/mL ampicillin. Cultivations for plasmid rescue with liquid LB media were prepared with 25 g/L LB and supplemented with 100 μg/mL ampicillin. 

The Aspergilli strains used in this study are presented in Table 1. All Aspergilli were cultivated on standard solid glucose-based minimal medium (MM) (1% glucose, 1 × nitrate salt solution, 0.001% Thiamine, 1 × trace metal solution, 2% agar), supplemented when required with 10 mM uridine (Uri), 10 mM uracil (Ura), and/or 4 mM L-arginine (Arg). Transformation media (TM) was prepared as MM media, apart from glucose, which was replaced with 1 M sucrose. Media for two-layer plating was freshly prepared and depending on the layer 75 µg/mL or 300 µg/mL hygromycin was added. 

### 2.2. PCR and Assembly of Plasmids by USER Cloning

All primers were obtained from Integrated DNA Technologies (IDT) and their sequences can be found in Table S1. PCR fragments for cloning were amplified in 35 cycles using proof-reading Phusion U Hot Start DNA Polymerase (Thermo Fisher Scientific) according to the instructions of the supplier. Standard reaction volumes were 50 μL containing 25 μL Phusion U Hot Start PCR Master Mix, 0.5 μM primers, 10–50 ng plasmid template and MilliQ water to reach the desired final volume. All vectors (see Table S2) were assembled by USER cloning [29].

Diagnostic PCR reactions were performed using DNA from conidia as template and MyTaq™ Plant-PCR Kit (Bioline). Prior to PCR reaction, spore suspensions were prepared by adding spores into 20 μL MilliQ water. The resulting samples were microwaved at 800 Watts for 3 min. Standard reaction volumes for diagnostic PCRs were 20 μL including 0.5 μM primers, 10 μL MyTaq™ Plant-PCR Kit, 3 μL of spore suspension and MilliQ water to reach the final volume. 

The *ErCas12a* gene from *Eubacterium rectale* [20] was codon-optimized for translation in *A. niger* and synthetized by GeneArt™ (Thermo Fisher Scientific). USER compatible backbone encoding the *ErCas12a* gene was created by fusing it with *A. nidulans tef1* promoter and terminator and then by incorporating into a digested USER vector backbone. The primer fusing the *tef1* promoter with the digested backbone included a new USER restriction site (PacI/Nt.BbvCI USER cassette). A total of six Mad7-CRISPR vectors with different selective markers: pDIV298 (*pyrG,* selection/counterselection by uracil + uridine prototrophy/5-fluoroorotic acid), pDIV299 (*argB*, selection by arginine prototrophy), pDIV300 (*hygB,* selection by hygromycin resistance), pDIV301 (*ble*, selection by bleomycin resistance), pDIV302 (*nat*, selection by nourseothricin resistance) and pDIV303 (*amdS,* selection/counterselection by acetamide prototrophy/fluoroacetamide) were constructed by inserting a PCR fragment containing the P*tef1*::ErCas12a::T*tef1* into USER cassette of pAC572, pAC573, pAC574, pAC575, pDIV296 and pDIV297, respectively (see Table S2). The PCR fragment containing the *ErCas12a* gene was generated by primers PR_DIV1503 and PR_DIV1504 using the synthetic gene from GeneArt™ as a template. Newly created Mad7-CRISPR USER compatible backbones were used in subsequent experiments to create vectors encoding the gRNA expression cassette. The gRNA cassette was constructed fusing two PCR products by USER fusion, see Figure S1.

The *mRFP* gene-targeting PCR fragments for *A. nidulans* and *A. niger* were created with primers containing overhangs at the 5′ ends homologous to *yA (*PR_DIV3219- PR_DIV3220 and *albA (*PR_DIV3217- PR_DIV3218), respectively (See Table S1). In case of *A. campestris*, we used a plasmid containing a 54 bp synthetic CRISPR-targeting site (CTS) covering multiple Mad7, Cas9 and Cpf1 PAM sequences, flanked by 2 kb homology arms upstream and downstream of the *ku70* locus (PR_DIV3073-PR_DIV3074 and PR_DIV3075-PR_DIV3076). In case of *A. oryzae,* a plasmid containing the same short linker as described above for *A. campestris* flanked by 1.4 kb homology arms upstream and downstream of the *ku70* locus. This plasmid was used as PCR template to amplify to fragments and both fragments have 700 bp overlapping region (PR_DIV3091-PR_DIV3092 and PR_DIV3093-PR_DIV3094).

### 2.3. Transformation and Strain Validation by Diagnostic PCR

Protoplasts were generated as described by Nielsen et al. [26]. For transformation, approximately 10^7^ protoplasts and 1 μg of Mad7-CRISPR-tRNA vector were mixed with 150 μL PCT solution and incubated for 10 min at room temperature, followed by addition of 250 μL of ATB and plating on TM with selection. 

TM plates were incubated at 37 °C (*A. nidulans*) or at 30 °C (*A. oryzae* and *A. campestris*). Transformations of *A. niger* were performed using a two-layer plating procedure and in some cases two different temperatures; for more details see section “Two-phase protocol” below. In gene-editing experiments, we added either 1 μg of oligonucleotides (IDT, see Table S1) or 1 μg of *mRFP* gene-targeting PCR fragment as RT, together with 1 μg of Mad7-CRISPR-tRNA vector. For *A. campestris*, the deletion cassette was liberated and linearized from pDIV708 digestion with SwaI, and 1 μg was used for transformation together with 1 μg of CRISPR Mad7 vectors. For *A. oryzae*, 0.7 μg of each PCR fragment (see Section 2.2) was used as template together with 1 μg of Mad7-CRISPR-tRNA vector.

Target-specific genome engineering was analyzed by diagnostic PCR. Primers for detecting precise gene-editing mutations were designed to anneal up- and downstream from the gene-targeting sequence or within the PCR construct. The amplified bands for gene-targeting experiments with oligonucleotides were subsequently purified with Illustra GFX PCR DNA and Gel Band Purification Kit (GE Healthcare Bio-Sciences) and sent for sequencing (StarSEQ).

### 2.4. Two-Phase Protocol

The procedure includes a first phase favoring Mad7 activity and a second phase favoring fungal recovery. Specifically, a mixture of protoplasts and DNA is incubated on ice for 15 min. Next, 1 mL PCT is added, and the mixture is then incubated for 15 min at room temperature. After incubation, 13 mL of TM with 75 µg/mL hygromycin for *A. niger* and 50 µg/mL for *A. campestris* is gently added to the mix. The total volume is then poured into an empty Petri dish. This first layer is allowed to solidify before the plate is incubated at 37 °C. Other incubation temperatures may be used as controls; see Section 3.4. The second TM layer, containing higher hygromycin concentrations (300 µg/mL for *A*. *niger* and 100 µg/mL hygromycin for *A. campestris*), is added the next day. Plates are then incubated at the temperature optimum of the fungus as well as at a control temperature; see Section 3.4. On the third day, all plates including controls are incubated at a temperature favorable for growth of the target fungus for approximately 3–4 days for *A. niger* and 7–10 days for *A. campestris*.

### 2.5. Fluorescence Photography

Red fluorescence was examined using our previously described in-house build digital camera setup [30]. Images of agar plates with either *A. nidulans* or *A. niger* colonies were captured using an SLR camera (Nikon D90) equipped with Light and Filter set (NIGHTSEA™). Camera settings for fluorescent images were as follows: ISO Speed—ISO250, F-stop—f/7.1, Exposure time—1.6 s, Focal length—60 mm. Images were saved in JPEG format with a 300 dpi resolution.

## 3. Results

### 3.1. A Versatile Mad7 System to Facilitate CRISPR-Based Gene Editing

To enhance functionality in fungi, our Mad7-CRISPR vectors contain a *mad7* gene, which is codon-optimized for expression in *A. niger* and extended by a sequence encoding the SV40 nuclear localization signal (PKKKRKV) to ensure nuclear transport of Mad7. The strong constitutive *A. nidulans tef1* promoter and terminator [31,32] were selected to control *mad7* expression. In our system (Figure 1B), the gRNA is released from a single glycine tRNA-based splicing-cassette [33] from a construct controlled by the *A. fumigatus* U3 polymerase III promoter and terminator, which we have previously employed for this purpose [15]. 

### 3.2. Mad7 Efficiently Mediates Template-Directed Mutagenesis and Gene Disruption in A. nidulans

**Figure 2:**
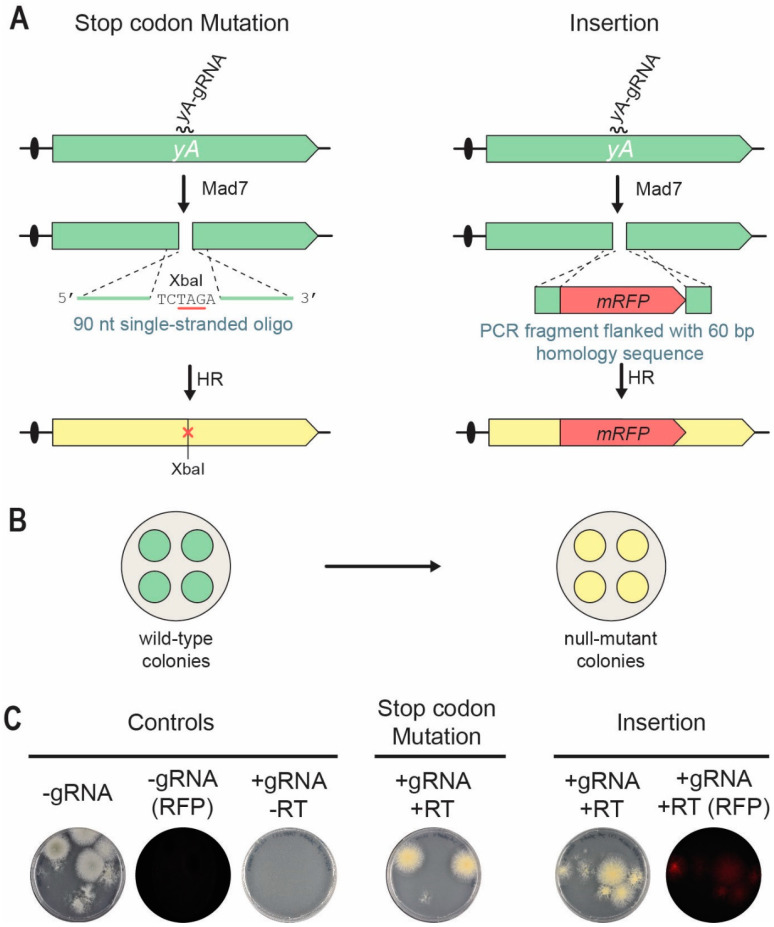
Mad7-induced gene editing of the *yA* locus in *A. nidulans*. (**A**) Strategies for introducing a specific stop-codon/XbaI mutation (left) and an *mRFP* insertion (right) into *yA*. A DNA DSB is introduced in *yA* by the Mad7/*yA*-gRNA CRISPR nuclease. A single-stranded oligonucleotide is used as RT to direct introduction of stop-codon/XbaI mutation into *yA* by homologous recombination (HR). A PCR fragment is used as RT to direct insertion of *mRFP* into *yA* by HR. See main text for further details. (**B**) Mutation of *yA* changes the conidia color of transformants growing on solid medium from wild-type green to yellow. (**C**) To the left, transformation controls with empty Mad7-CRISPR plasmid (pDIV298) images in visible or in red fluorescent light (RFP); and with the *yA*-Mad7-CRISPR plasmid (pDIV711) in the absence of a RT. In the middle, introduction of the stop-codon/XbaI mutation into *yA* by co-transforming *A. nidulans* with pDIV711 and a single-stranded oligonucleotide (PR_DIV3197) serving as RT. RT mediated repair introduces an XbaI site and an amber stop codon. To the right, insertion of *mRFP* into *yA* by co-transforming *A. nidulans* with plasmid (pDIV711) and a *mRFP*-PCR fragment serving as RT.

Since the native producer of Mad7, *Eubacterium rectale*, is a prominent human gut symbiont growing at 37 °C, we reasoned that Mad7 may show higher activity at this temperature. Indeed, Mad7 gene-editing experiments in *Bacillus subtilis* were performed at 37 °C [34]. In a first test of our Mad7-CRISPR system for genetic engineering of filamentous fungi, we selected the fungus *A. nidulans* as it prefers to grow at 37 °C. Specifically, we chose the NHEJ-deficient strain NID1 (see Table 1) and investigated whether Mad7 would be able to stimulate site-directed mutagenesis and gene disruption/insertion. Accordingly, using pDIV298 as backbone, we constructed a Mad7-CRISPR vector, pDIV711, which encodes a gRNA targeting the *yA* gene [15,35]. Inactivation of *yA* causes the normally green conidia of *A. nidulans* to turn yellow (Figure 2B), a phenotype that is easy to score [36]. To explore the possibility of using Mad7 for gene editing, we co-transformed *A. nidulans* protoplasts with pDIV711 and a RT. For site-directed mutagenesis, we used a 90 nt (nucleotides) single-stranded oligonucleotide (PR_DIV3197), which bridges the potential Mad7-induced DNA DSB as RT. This oligonucleotide is able to introduce an XbaI site and an amber stop codon in *yA* that disrupts its function [30], see Figure 2A. For introduction of *mRFP* into *yA*, a PCR fragment was used as RT. This fragment contained *mRFP* flanked by 60 bp of up- and 60 bp of downstream sequences relative to the position of the DNA DSB see Figure 2A. Protoplasts were plated on solid TM medium containing arginine for selection of pDIV711. Control experiments where *A. nidulans* was transformed with an empty Mad7-CRISPR vector or with the same amounts of a *yA*-Mad7-CRISPR vector in the absence of a RT produced ten and zero, transformants, respectively (see Figure 2C). This result indicates that Mad7 guided by the *yA*-gRNA is able to make lethal DNA DSBs in the NHEJ-deficient *A. nidulans* strains. In agreement with this, transformants were recovered when pDIV711 was co-transformed with either the oligonucleotide or the *mRFP-*PCR fragment as RT. The observation that lethal CRISPR nuclease-induced breaks are rescued in the presence of an appropriate RT is indicative of a successful gene-editing experiment, and we have previously called this a TAPE test [37]. Indeed, in the presence of the repair oligonucleotide, all transformants were yellow; and in the presence of the *mRFP*-PCR fragment, all transformants were, unlike the control strains, able to emit red fluorescent light. Two additional trials for each experiment were performed with similar results (see Figures S2 and S3). For each experiment and for each trial, one transformant was randomly selected for further analysis. PCR fragments covering the mutated regions were sequenced; and in all six cases, the expected mutation/*mRFP* sequence was introduced without any other accompanying alterations at the target locus. Hence, Mad7 can be efficiently used for template-directed mutagenesis and gene insertion in an NHEJ-deficient *A. nidulans* strain.

### 3.3. Mad7 Can Be Used for Marker-Free Gene Deletion in NHEJ-Proficient A. oryzae and A. campestris

CRISPR experiments for specific genetic engineering are most efficiently conducted in NHEJ-deficient strains [15]. However, in many cases, only NHEJ-proficient strains may be available as a starting point. In these cases, and especially if the planned experimental work includes introduction of more than a few genetic alterations, it is advisable to invest time and create a mutation in the NHEJ pathway [38]. Cas9 has often been used to prepare NHEJ-deficient and wild-type strains for future CRISPR experiments [39] and we therefore tested whether Mad7 could also serve as a CRISPR nuclease for this purpose.

To investigate this possibility, we explored whether it would be possible to delete the *ku70* gene in a *pyrG*Δ *A. oryzae* strain (ORY2) and in wild-type *A. campestris* using Mad7. Accordingly, and for each species, we constructed a Mad7-CRISPR vector encoding two gRNAs targeting the *ku70* open reading frames close to the start and stop codons, pDIV707 and pDIV709 (Table S2). 

Co-transformations of *A. oryzae* and *A. campestris* with the appropriate Mad7-CRISPR vector (pDIV707 or pDIV709) and gene-targeting substrate were performed at 30 °C. The gene-targeting substrates for deleting *ku70* were either a linearized cassette from the digestion of pDIV708 with SwaI (*A. campestris*), or, a PCR-based gene-targeting substrate (*A. oryzae*), see Materials and Methods. These experiments produced three and five colonies respectively; and each colony was purified and tested by diagnostic PCR. These analyses identified two *A. oryzae* and one *A. campestris* colonies, which contained a *ku70* deletion. Subsequent sequence analysis demonstrated that in all three cases gene deletion was performed by using the gene-targeting substrate as RT, as the 54 bp linker sequence had replaced the *ku70* coding sequences. Hence, Mad7 can be used to introduce specific NHEJ mutations in NHEJ-proficient Aspergilli.

### 3.4. Site-Directed Mad7-Induced Gene Mutation in A. niger

**Figure 3:**
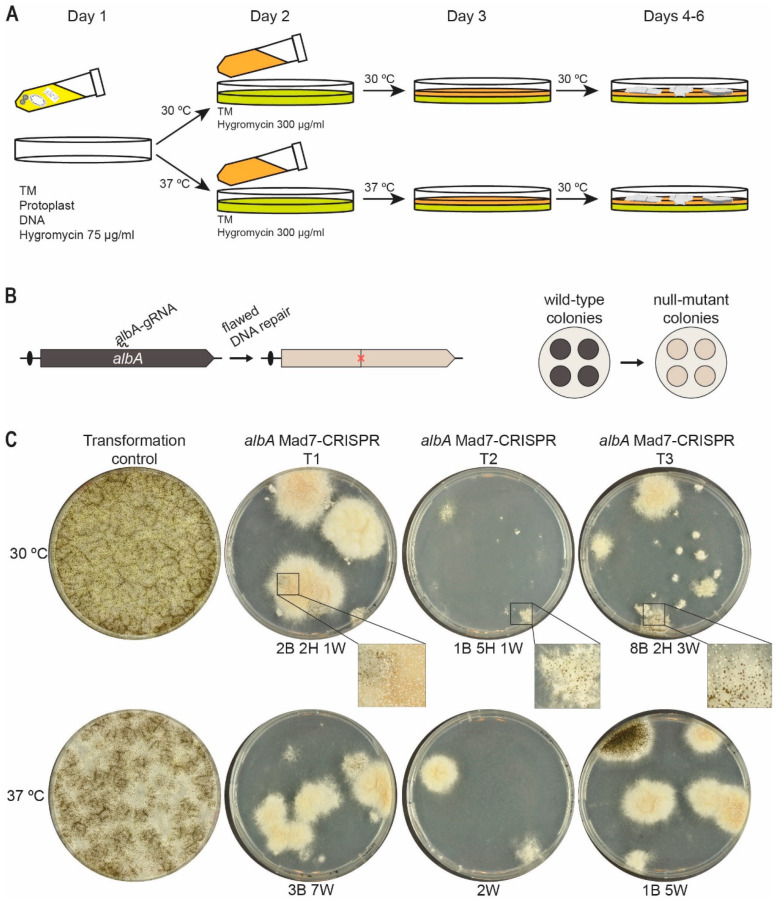
Efficient Mad7-induced mutagenesis in *A. niger*. (**A**) Scheme illustrating the two-phase transformant recovery scheme. Protoplasts transformed with a Mad7-CRISPR plasmid are mixed with melted medium containing low stringency hygromycin concentration and plated as the first layer; see Materials and Methods for details. When media solidifies, plates are incubated at 30 °C favoring growth of *A. niger,* or 37 °C favoring Mad7 activity. After one day of incubation, overlay medium containing high-stringency hygromycin concentration is added to form the second layer, and plates are then transferred for further incubation at the temperature indicated. (**B**) Mad7-induced mutagenesis of *A. niger albA* at a specific position indicated by a red X (left) and phenotypic consequence of mutation of colonies, which changes conidia color from wild-type black to mutant *albA* white (right). (**C**) Experiment showing Mad7-induced mutagenesis of *albA*. Transformation of protoplasts using the empty Mad7-CRISPR plasmid (indicated as control) or with the *albA* Mad7-CRISPR vector in three independent trials (indicated as T1–T3). All experiments were plated at two different temperatures as indicated. For trials T1–T3, numbers of transformants with specific phenotypes are indicated below plates: B, Black; H, Heterokaryon (black and white); W, White.

Since many organisms preferably propagate at temperatures other than 37 °C, different temperatures for Mad7-based gene editing have been employed depending on the organism. For example, in zebrafish, genome editing with Mad7 required a heat-shock of 34 °C to efficiently perform its activity [40]. Moreover, Lin et al. [41] achieved promising results in Japonica rice (*Oryza sativa*) performing gene-editing events at 26 °C indicating that a broader temperature range can be applicable for Mad7 gene editing despite that it may not act with optimal activity. This is important, as many fungi often grow at temperatures lower than 37 °C. In a next step in our efforts to make a versatile Mad7-based gene-editing system, we therefore investigated whether *A. niger*, which prefers 30 °C, could be efficiently engineered by Mad7. Since the optimal temperatures for transformation of *A. niger* and Mad7 activity do not appear to be the same, we envisioned that gene-editing success-rates could be increased by applying a two-phase transformant recovery scheme, see Figure 3A. In this way, it is possible to use a different temperature and selection pressure during a first incubation phase, which favors Mad7 activity and where a low selection pressure is applied; and a subsequent phase, which favors the growth condition of *A. niger* and where a higher selection pressure is applied.

In a first attempt to investigate Mad7 activity in *A. niger*, we constructed a Mad7-CRISPR vector (pDIV313) designed to introduce mutations into *albA* in an NHEJ-proficient *A. niger* strain [15], NIG1, via flawed NHEJ repair of Mad7-induced DNA DSBs. In this assay, we exploit that *albA* gene encodes a polyketide synthase responsible for the formation of the characteristic black pigment of the conidia of *A. niger* [42] and mutant strains are therefore easily scored as they develop white conidia (Figure 3B). Using this setup, NIG1 was transformed in triplicate with either the empty CRISPR-Mad7 plasmid pDIV300 coding only for Mad7, or with the CRISPR-MAD7 plasmid pDIV313, which encodes a gRNA targeting the *albA* gene. The experiment was performed using the two-phase transformant recovery scheme outlined above (see also Materials and Methods for the protocol). Hence, in the first phase, two plating steps with increasing selection pressure are performed at either 30 °C (favoring *A. niger* growth) or 37 °C (favoring Mad7 activity). In the second phase, the plates were incubated at 30 °C to allow an optimal growth temperature for *A. niger* (Figure 3A).

As expected, transformation with pDIV300, using both 30 °C and 37 °C in the first phase, generated a large number of transformants as it only produces the Mad7 apoenzyme that cannot locate and cleave the target sequence. In contrast, co-transformation with pDIV313 encoding Mad7 and the *albA* gRNA, and using the same two temperatures through the first phase, produced only few transformants despite that similar amounts of DNA were used in the two experiments. These results indicate that the Mad7-*albA* gRNA complex efficiently cleaves the *albA* locus to reduce survival of the transformants. In agreement with this, most transformants produced white conidia indicating that mutations have been introduced into the *albA* locus due to Mad7-induced breaks followed by defective NHEJ repair. Lastly, by inspecting the transformants obtained using either 30 °C or 37 °C in the first phase, we noticed that the transformants obtained using 30 °C were often heterokaryons displaying a black/white phenotype, whereas those obtained using 37 °C were typically solid white or black, see Figure 3C. These results indicate that a more robust Mad7 activity is obtained by using 37 °C in the first incubation phase.

### 3.5. Mad7 Efficiently Mediates Template-Directed Mutagenesis and Gene Disruption in A. niger

Encouraged by the efficient Mad7-induced mutagenesis of *albA* in *A. niger*, we next investigated whether it would be possible to introduce template-directed mutations in *albA* in *A. niger* via selection-free gene-editing events. For this purpose, we chose the NHEJ-deficient strain, NIG96, which contains a deletion of *kusA* to prevent formation of transformants generated from NHEJ pathway.

**Figure 4:**
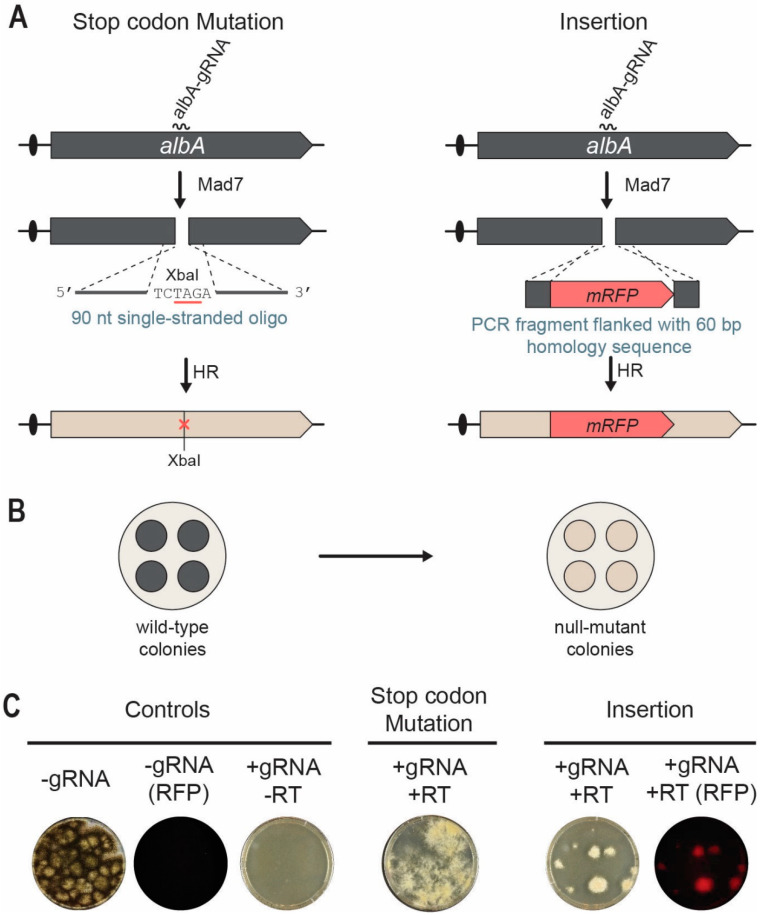
Mad7-induced gene editing of the *albA* locus in *A. niger*. (**A**) Strategies for introducing a specific XbaI site/amber stop codon into *albA* (left) and an *mRFP* insertion (right) into *albA* locus. A DNA DSB is introduced in *albA* locus by Mad7 containing an *albA* specific gRNA. A single-stranded oligonucleotide is used as RT to direct introduction of the XbaI site/amber stop codon by HR. A PCR fragment is used as RT to direct insertion of *mRFP* into *albA* by HR. See main text for further details. (**B**) Mutation of *albA* changes the conidia color of transformants growing on solid medium from wild-type black to white. (**C**) To the left, transformation controls with empty Mad7-CRISPR plasmid (pDIV300) imaged in visible and red fluorescent light as indicated; and with the *albA*-Mad7-CRISPR plasmid (pDIV313) in the absence of a (RT). In the middle, introduction of a stop-codon mutation into *albA* using the *albA* Mad7-CRISPR plasmid (pDIV313) and an oligonucleotide (PR_DIV3196)-based RT. To the right, insertion of *mRFP* into *albA* using the *albA*-Mad7-CRISPR plasmid (pDIV313) and the PCR-based RT. Plates were imaged with visible and red fluorescent light as indicated.

We first investigated whether a single-stranded oligonucleotide could serve as a RT and therefore used to edit *albA*. Accordingly, we co-transformed *A. niger* in triplicates with pDIV313 and oligo PR_DIV3196 (see Table S1). Successful repair by the 90 nt oligonucleotide will introduce an XbaI site/amber stop codon into *albA* to generate white colonies (Figure 4A,B). After transformation, the protoplasts were plated according to the two-layer method using 37 °C in the two first incubation steps, and 30 °C for recovery and growth. In parallel, NIG96 was also transformed with the empty Mad7 CRISPR plasmid pDIV300. As expected for a robust cutting/repair reaction, the number of transformants were much higher in the presence of the oligonucleotide RT as compared to the numbers obtained in the absence of the oligonucleotide RT, see Figure 4C. In agreement with this, all transformants obtained in the presence of the oligonucleotide RT produced white conidia.

With the aim of inserting *mRFP* and disrupting *albA*, NIG96 was next co-transformed with pDIV313 and a PCR-based *albA* gene-targeting substrate containing *mRFP* flanked by 60 bp of up- and downstream *albA* sequences (Figure 4A). After transformation, we used the two-phase protocol using 37 °C and 30 °C for incubation in the first phase. In contrast to the control experiment where no transformants were achieved in the absence of a RT (Figure 4C), several transformants were obtained in the presence of the *mRFP*-PCR gene-targeting-substrate. In agreement with efficient Mad7-assisted gene targeting, all transformants emitted red fluorescent light. Two additional trials for each experiment were performed with similar results (see Figures S4 and S5). For each experiment and for each trial, one transformant was randomly selected for further analysis. PCR fragments covering the mutated regions were sequenced, and in all six cases, the expected mutation/*mRFP* sequence was introduced. Together, our data show that Mad7-induced DNA DSBs can be efficiently repaired by using single-stranded oligonucleotides and PCR fragments containing short targeting sequences to introduce desirable genome modifications in *A. niger*.

## 4. Conclusions

We have demonstrated that Mad7, an IP friendly CRISPR nuclease, can be used to perform gene editing in four different *Aspergillus* species: the genetic model fungus (*A. nidulans*), two industrial workhorses (*A. niger* and *A. oryzae*) and a true wild-type isolate (*A. campestris*). Hence, we envision that Mad7 can be broadly used to perform gene editing in fungi. *A. niger* was investigated in more detail as we used Mad7 to introduce several different types of gene edits such as unspecific and specific template-directed mutagenesis. For the latter, we demonstrated the efficient introduction of small mutations as well as gene insertion into the pigment gene *albA* via short targeting sequences. These observations open up promising avenues for cell factory construction using Mad7. Similarly, the fact the Mad7 was successfully used to engineer *A. campestris*, a species that has not previously been genetically engineered, suggests that Mad7 can also be used in future enzyme and secondary metabolite discovery endeavors.

## Figures and Tables

**Table 1:** Fungal strains used in this study.

Species	Strain Name	ID Number	Genotype	Reference
*A*. *nidulans*	NID1	IBT29539	*argB2, pyrG89, veA1, nkuA*Δ	[26]
*A*. *niger*	NIG1	ATCC1015		
*A*. *niger*	NIG96		*pyrG1, kusA*Δ	[15]
*A. oryzae*	ORY2	RIB40	*pyrG89*	[27]
*A. campestris*		IBT28561		[28]

## Data Availability

Not applicable.

## Supplementary Materials

The following supporting information can be downloaded at: https://www.mdpi.com/article/10.3390/jof9010016/s1, Table S1: List of primers; Table S2: List of plasmids; Figure S1: Primer design for construction of gRNA expression cassettes; Figure S2: Single-stranded oligonucleotide-mediated site-specific mutagenesis in *A. nidulans*; Figure S3: Mad7-induced gene insertion in *A. nidulans*; Figure S4: Single-stranded oligonucleotide-mediated site-specific mutagenesis in *A. niger*; Figure S5: Mad7-induced gene insertion in *A. niger.* [43,44,45,46] in Supplementary Materials.
