# Supplementary material for: A Mad7 System for Genetic Engineering of Filamentous Fungi
Source: J Fungi (Basel). 2022 Dec 22;9(1):16. doi: 10.3390/jof9010016 (PMC9865164; doi:10.3390/jof9010016)
Supplement: Supplementary file 1 [file jof-09-00016-s001.zip › jof-2098407-supplementary.pdf]

Supplementary Table S1: List of primers

Primer ID	Description[A]	Sequence 5′→3′
**Primers for Mad7 cloning**		
PR_DIV1462	ANPtef-F_PacI regen	GGGTTTAAUGCTGAGGGTTTAATTA AGACCTCAGCCGAGACAGCAGAAT CACCG
PR_DIV0308	Ptef-RU	ATGGTGAAGGUTGTGTTATGTTTTG
PR_DIV1503	MAD7_ANIGopt-NLS_F	ACCTTCACCAUATGAACAACGGCAC CAACAAC
PR_DIV1504	MAD7_ANIGopt-NLS_R	ATGTCCGCUTCAGACCTTGCGCTTCT TCTT
PR_DIV0307	Ttef-FU	AGCGGACAUTCGATTTATGC
PR_DIV0022	Ttef-mRFP-RU-PacI Dw	GGTCTTAAUGTATTGGGATGAATTTT GTATGC
**Primers for gRNAs**		
PR_DIV1507	Afum-P U3_F-PacI Up	GGGTTTAAUGATCACATAGATGCTC GGTTGAC
PR_DIV1508	Afum-T U3_R-PacI Dw	GGTCTTAAUACCCTGAGAAGATAGA TGTGAATG
PR_DIV1505	Afum-P U3_R-long	ACAAGAGUAGAAATTAAAAAGGTC TTTTGACTGCATCATCCGTGAATCGA AC
	Afum-P U3_R-short	ACAAGAGUAGAAATTAAAAAGGTC TTTTGAC
PR_DIV1550	*A.niger* ATCC1015 PS_MAD7 *albA*-F	ATGCTTCCAUGCAATTGCATCATTGG TCTAGTGGTAGAATTC
PR_DIV1551	*A.niger* ATCC1015 PS_MAD7 *albA*-R	ATGGAAGCAUTGCTGATCTACAAGA GTAGAAATTAAAAAGGTC
PR_DIV3119	*A. nidulans yA* gRNA2 Mad7	ACTCTTGUAGAT ATTGGCGGCGCTGCGCAAAAG GCATCATTGGTCTAGTGGTAGAATTC
PR_DIV3073	*A.campestris* IBT28561 PS1_MAD7 Ku70-R	ACTCGTTGATCUCAATGGATCTACA AGAGTAGAAATTA
PR_DIV3074	*A.campestris* IBT28561 PS1_MAD7 Ku70-F	AGATCAACGAGUCGAGCATCATTGG TCTAGTGGTA
PR_DIV3075	*A.campestris* IBT28561 PS2_MAD7 Ku70-R	ATGAAGAGUCGCCGGGAGAATCTA CAAGAGTAGAAATTA
PR_DIV3076	*A.campestris* IBT28561 PS2_MAD7 Ku70-F	ACTCTTCAUCGGCATCATTGGTCTAG TGGTA
PR_DIV3086	*A.oryzae* RIB40 PS1_MAD7 Ku70-R	ATCGCTGACUTCGATAGATCTACAA GAGTAGAAATTAAAAAGG
PR_DIV3087	*A.oryzae* RIB40 PS1_MAD7 Ku70-F	AGTCAGCGAUTCGAGCATCATTGGT CTAGTGG
PR_DIV3088	*A.oryzae* RIB40 PS2_MAD7 Ku70-R	AAACATTAUCTGCATCTACAAGAGT AGAAATTAAAAAGG
PR_DIV3089	*A.oryzae* RIB40 PS2_MAD7 Ku70-F	ATAATGTTUCTCTATCCGCATCATTG GTCTAGTGG
**Primers for rescue templates**		
PR_DIV3217	*albA*_gRNA1_RFP cassette-F	TCGAAGCTGGCCTGCGCCGTCTGCTC CAAGCGAAGAATAGTACCATTGTCC AGTCCTTTT ATTCCCTTGTATCTCTACACACAGG
PR_DIV3218	*albA*_gRNA1_RFP cassette-R	ACGATGCTCGTGAAGCGTGGGAAGA GCTTCCGATGAGACGGCGGGAGCTT

		CGCGATCTCT TCTTAATGCGATGCTTCCATTGCC
PR_DIV3219	*yA*_gRNA2_RFP cassette-F	GTGTCGAGCCCCCGGTGATCTACGT CGATCCCGAATTCAACGGTTGGGTC AGCCTTAATT ATTCCCTTGTATCTCTACACACAGG
PR_DIV3220	*yA*_gRNA2_RFP cassette-R	TCAACAAACTGTCCGTCAACTTCAT ATACCCACATTGGGTGATTGTCGAC AGAAAAAGTT TCTTAATGCGATGCTTCCATTGCC
PR_DIV3077	Ku70-Deletion cassette ACAM-F1	GGGTTTAAUCCCTGTATATTTCCGAC CTACG
PR_DIV3078	Ku70-Deletion cassette ACAM-R1	AAACCCTUGGGCTCGATATCGGCAC AGAAAACGGAGCAGAGCCTCTCG
PR_DIV3079	Ku70-Deletion cassette ACAM-F2	AAGGGTTUCGCCCTTCCCATCTCAA GATGCGGCCTTCACCTTATGATTGCT ACATACC
PR_DIV3080	Ku70-Deletion cassette ACAM-R2	GGTCTTAAUCAAGATCAAGTGCTGC GATATG
PR_DIV3091	Ku70-Deletion cassette AORY-F1	GGGTTTAAUCTCCAATGTCCGCGTAT TGC
PR_DIV3092	Ku70-Deletion cassette AORY-R1	AAACCCTUGGGCTCGATATCGGCAC AGAAACATCTTTCACTGTTTTGTATC CC
PR_DIV3093	Ku70-Deletion cassette AORY-F2	AAGGGTTUCGCCCTTCCCATCTCAA GATGCGGCAAATGTGTACATGTTCG AGAT
PR_DIV3094	Ku70-Deletion cassette AORY-R2	GGTCTTAAUCACAATCGCTTTCCTCC ATG
PR_DIV3095	Ku70-Deletion cassette AORY-F3	CTCCAATGTCCGCGTATTGC
**Primers for diagnostic PCR**		
PR_DIV1555	MAD7_ANIG Opt_Seq1-F	CTCCCGCTTCGCCACCTC
PR_DIV1556	MAD7_ANIG Opt_Seq1-R	GAGGTGGCGAAGCGGGAG
PR_DIV1557	MAD7_ANIG Opt_Seq2-F	CAACTACAACGGCTACAACCTC
PR_DIV1558	MAD7_ANIG Opt_Seq2-R	GAGGTTGTAGCCGTTGTAGTTG
PR_DIV1559	MAD7_ANIG Opt_Seq3-F	CTACGTCACCCAGAAGCCTTAC
PR_DIV1560	MAD7_ANIG Opt_Seq3-R	GTAAGGCTTCTGGGTGACGTAG
PR_DIV1561	MAD7_ANIG Opt_Seq4-F	CAACGACAACCTCCACACCA
PR_DIV1562	MAD7_ANIG Opt_Seq4-R	TGGTGTGGAGGTTGTCGTTG
PR_DIV1563	MAD7_ANIG Opt_Seq5-F	TCGCAAAGAGTGGAAAGAGAT
PR_DIV1564	MAD7_ANIG Opt_Seq5-R	ATCTCTTTCCACTCTTTGCGA
PR_DIV1565	MAD7_ANIG Opt_Seq6-F	GACACCATCGACATCACCAAG
PR_DIV1566	MAD7_ANIG Opt_Seq6-R	CTTGGTGATGTCGATGGTGTC
PR_DIV3221	int RFP-Seq-F	TACATGGCCAAGAAGCCCGTG
PR_DIV3222	int RFP-Seq-R	CTTGAAGCGCATGAACTCC
PR_DIV3223	ANPgpdA-Seq_F	CGAGCTTTCCCACTTCATCG
PR_DIV3224	ANtrpC-Seq-R	CTAAGCTATTCTTCTGCTTCGCC
PR_DIV0420	albA1443_seq_F	CATGTGTATAAAGTGTGCGTCTCAT
PR_DIV0421	albA1443_seq_R	GTGCAGCTCAGAACACCAGTG
PR_DIV0418	yA1442_seq_F	CGTCCTCGAAGGAACACATCT
PR_DIV0419	yA1442_seq_R	CTGATTGACATACGAGAGGATGG
PR_DIV1746	ANPgpdA_seq1_R	CCTCATGGCGATTGCAGTC
PR_DIV3081	ACAM ku70.deletion_Seq1-F	CTGTTTCCGTAGCATTTGTACTTCCT

PR_DIV3082	ACAM ku70.deletion _Seq2-F	GTCGTAGGAGTGATATGAGTAAATGAA
PR_DIV3083	ACAM ku70.deletion _Seq1-R	GGGAGTATTCGTCACAGAGCAG
PR_DIV3084	ACAM ku70.deletion _Seq2-R	AGTATACCTTGACACTCACGGT
PR_DIV3098	AORY ku70.deletion _Seq1-F	CACAATCGCTTTCCTCCATG
PR_DIV3100	AORY ku70.deletion _Seq1-R	CAATACCGCCCTCAACAAGG
PR_DIV3096	MAD7_AORY ku70_Seq1-F	GGCGCACTTTCAGGATTGAG
PR_DIV3097	MAD7_AORY ku70_Seq2-F	GACAGGCAGACACCTAGGAA
PR_DIV3099	MAD7_AORY ku70_Seq1-R	GAGCATGCATTTCTGGGATTAG
**Primers for oligonucleotides**		
PR_DIV3196	*albA*-gRNA1 oligo XbaI	CGTCTGCTCCAAGCGAAGAATAGTACCATTGTCCAGTCCTtctagaGAGATCGCGAAGCTCCCGCCGTCTCATCGGAAGCTCTTCCCACG
PR_DIV3197	yA-gRNA1 oligo XbaI	GAGAGAGTTAGCAGAAATACAGTACGCAGAAGATAATCCTTATtctagaCTTCGGCGGAGTATCATAACATCGAGGTTGAGTCTGGCTAT

[A]Color code: Annealing sequence, target sequence, homologous recombination sequence, PacI/Nt.BbvCI cassette, Mad7 direct repeat, custom USER overhangs, thymine-> uracil substitution

Supplementary Table S2: List of plasmids

Plasmid ID	Description	Reference
**Cloning vectors and PCR templates**		
P GeneArt™	Mad7 codon optimized for *A. niger*	
pAC572	pAMA1-pyrG-PacI/Nt.BbvCI	[14,30]
pAC573	pAMA1-argB-PacI/Nt.BbvCI	[14,30]
pAC574	pAMA1-hph-PacI/Nt.BbvCI	[14,30]
pAC575	pAMA1-ble-PacI/Nt.BbvCI	[14,30]
pDIV296	pAMA1-nat1-PacI/Nt.BbvCI	[43]
pDIV297	pAMA1-amdS-PacI/Nt.BbvCI	[43]
pDIV309	pAMA1-hph-MAD7-PAf_U3-AN_tRNA-MAD7 crRNA-PS-AN_tRNA-TAf_U3	[43]
pDIV708	*A. campestris ku70* deletion repair template – 2 kb homology regions	[43]
pDIV710	*A. oryzae ku70* deletion repair template – 1.4 kb homology regions	[43]
pAC1014	P*gpdA-mRFP*.T*trpC* repair template	
**pAMA1 with different markers and MAD7**		
pDIV298	pAMA1-pyrG-Mad7-PacI/Nt.BbvCI	[43]
pDIV299	pAMA1-argB-Mad7-PacI/Nt.BbvCI	[43]
pDIV300	pAMA1-hph-Mad7-PacI/Nt.BbvCI	[43]
pDIV301	pAMA1-ble-Mad7-PacI/Nt.BbvCI	[43]
pDIV302	pAMA1-nat1-Mad7-PacI/Nt.BbvCI	[43]
pDIV303	pAMA1-amdS-Mad7-PacI/Nt.BbvCI	[43]
**pAMA1-MAD7 vectors with gRNA constructs**		
pDIV313	pDIV313 pAMA1-hph-Mad7-PS *A. niger albA*	This study
pDIV707	pDIV707 pAMA1-hyg-Mad7-2PS *A. campestris ku70*	This study
pDIV709	pDIV709 pAMA1-pyrG-Mad7-2PS *A. oryzae ku70*	This study
pDIV711	pDIV711 pAMA1-pyrG-Mad7-1PS *A. nidulans yA*	This study

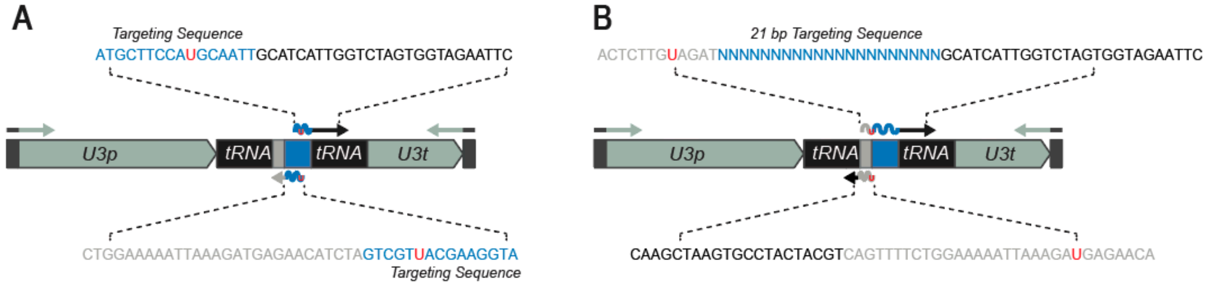

Supplementary Figure S1: Primer design for construction of gRNA expression cassettes. gRNA expression cassettes are assembled by fusion of two PCR fragments. Two strategies (out of many possible) are shown. (A) PCR fragments are fused via USER tail [29] overlaps present in the variable gRNA sequence. In this strategy, construction of each new gRNA expression cassette requires four primers, two that are constant primers (which can be recycled) and two that are variable as they contain the targeting sequence (which needs to be designed de novo for each new target site). The variable part of the primers overlap and the overlapping sequencing contains a uracil base (shown as small red U in primers). Hence, when the two PCR fragments are cut with USER enzyme mix (New England Biolabs) [44-46] that cuts at uracils, complementary tails are produced in the two PCR fragments, which can be used to fuse them in vivo by USER fusion in *E. coli*. The constant primers anneal to the ends of the gRNA expression cassette and are equipped with ends that are compatible with further USER cloning into a USER cassette present in the destination vector, see [29]. The two variable primers anneal to an upstream section of the tRNA (black) and to the constant part of the gRNA (gray), respectively. In both cases, variable gRNA sequences are present in the primer tails (in blue). In the example, we have used a target sequence in *albA* from *A. niger* . Note that positions that allow for formation of relevant USER overhangs in the two variable primer tails need to be identified for each new vector. The result of the fusion reaction is the assembly of entire gRNA expression cassette, including the desirable targeting sequence, which is compatible with a USER cassette in one of the destination vectors pDIV298-303. (B) Like in the previous strategy, construction of each new gRNA expression cassette requires four primers. However, three are constant primers (which can be recycled) and only one is variable as it contains the entire targeting sequence. Two constant primes are the same as those described above. The third constant primer anneal to tRNA sequence and most of the upstream constant part of the gRNA sequence for priming. The position of an uracil in the constant part of the gRNA sequence is indicated (small red U). The variable primer contains, the down-stream constant part of the gRNA, the entire variable part of the gRNA (which needs to be varied for each experiment) and the upstream tRNA sequence for priming. Note that in this example, we have indicated the variable region as a stretch of Ns. The position of the uracil in the constant gRNA region of the primer is indicated (small red U). USER fusion is used to merge the two PCR fragments into the gRNA expression cassette in a fragment, which is ready for further USER fusion into pDIV298-303. Both methods have been used in the present paper.

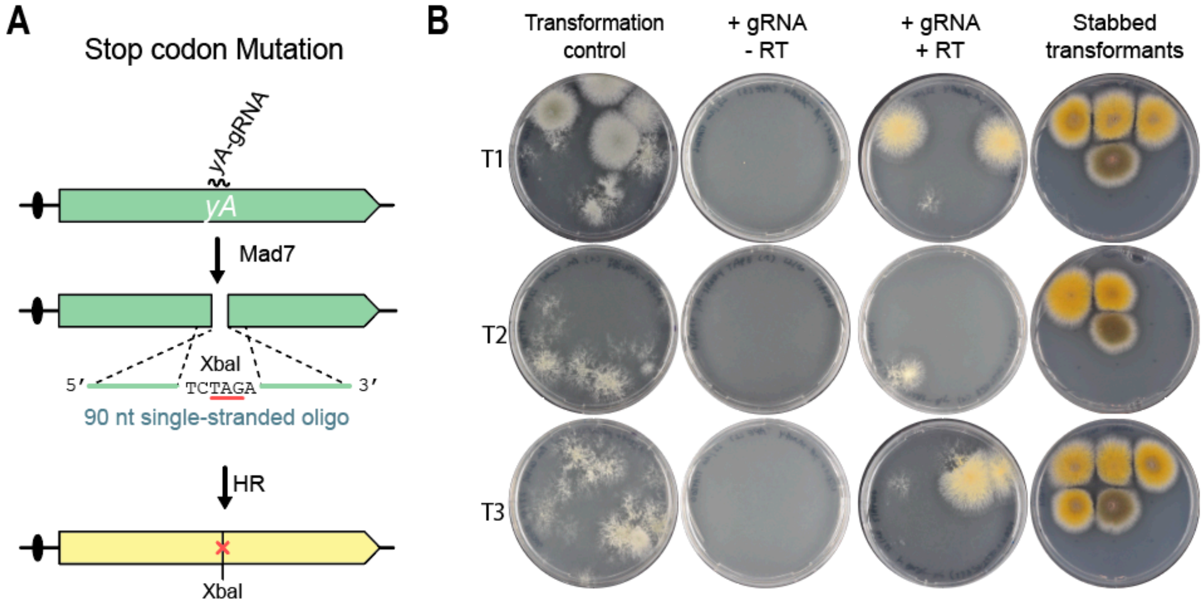

Supplementary Figure S2: Single-stranded oligonucleotide-mediated site specific mutagenesis in *A. nidulans*. A) Schematic representation of oligonucleotide mediated repair after Mad7 induced DNA double-strand-breaks in a given gene. B) Transformation results for all trials. Transformation control are protoplasts transformed with the empty Mad7-CRISPR plasmids pDIV298. Transformation results of *A. nidulans* protoplasts transformed with *yA*-Mad7 CRISPR vectors in the presence and absence of an oligonucleotide repair template as indicated. Stabs of independent transformants on a new plate are shown to the right with transformation control in the center of the plates

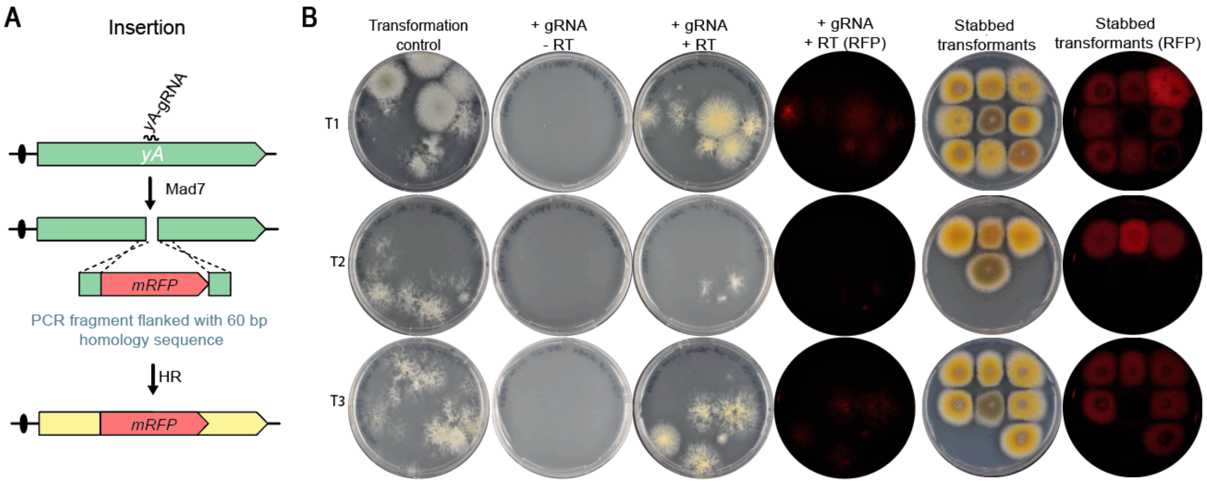

Supplementary Figure S3: Mad7 induced gene insertion in *A. nidulans*. A) Schematic representation of gene insertion experiments. Mad7 induces a DNA double-strand-break in the middle of a given gene. Subsequently, the DNA break is repaired by homologous recombination using a PCR fragment as repair template. In this case, the PCR fragment contains the RFP gene flanked by 60 bp of sequences identical to the two sides of the DNA break. B) Gene insertion in *yA* transformation results for all trials. Transformation control are protoplasts transformed with the empty Mad7-CRISPR plasmid pDIV298. Transformation results of *A. nidulans* protoplasts transformed with *yA*-Mad7 CRISPR vectors in the presence and absence of an RFP PCR fragment, which is used as repair template as indicated. Stabs of independent transformants on a new plate are shown to the right with the transformation control in the center of the plates

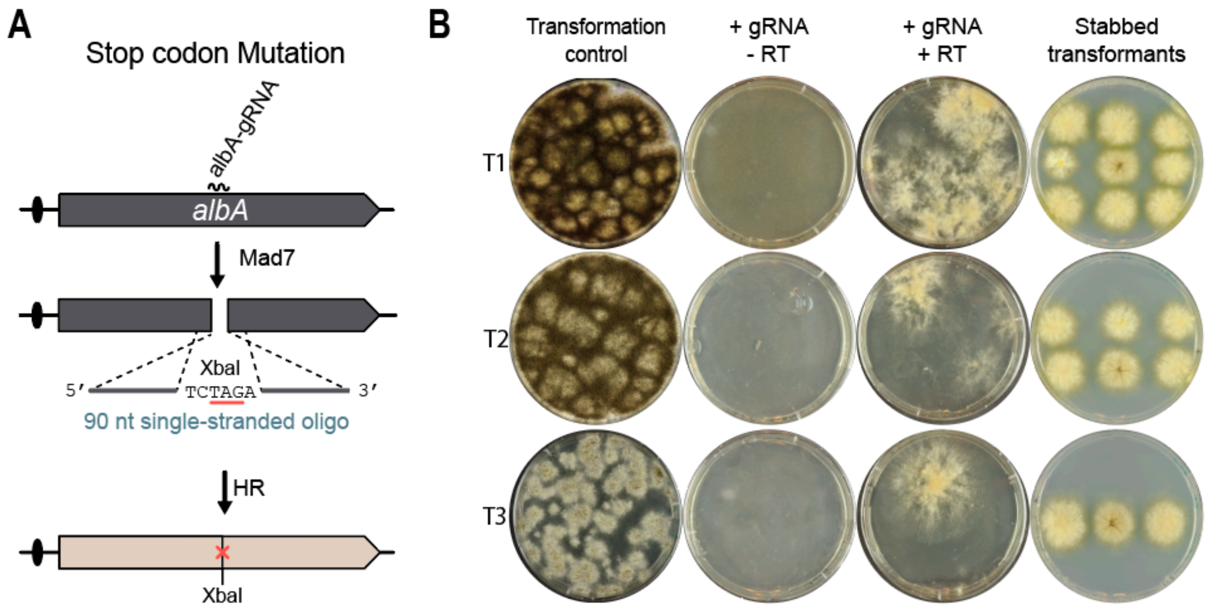

Supplementary Figure S4: Single-stranded oligonucleotide-mediated site specific mutagenesis in *A. niger*. A) Schematic representation of oligonucleotide mediated repair after Mad7 induced DNA double-strand-breaks in a given gene. B) Transformation results for all trials. Transformation control are protoplasts transformed with the empty Mad7-CRISPR plasmids pDIV300 for *A. niger*. Transformation results of *A. niger* protoplasts transformed with *albA*- Mad7 CRISPR vectors in the presence and absence of an oligonucleotide repair template as indicated. Stabs of independent transformants on a new plate are shown to the right with transformation control in the center of the plates

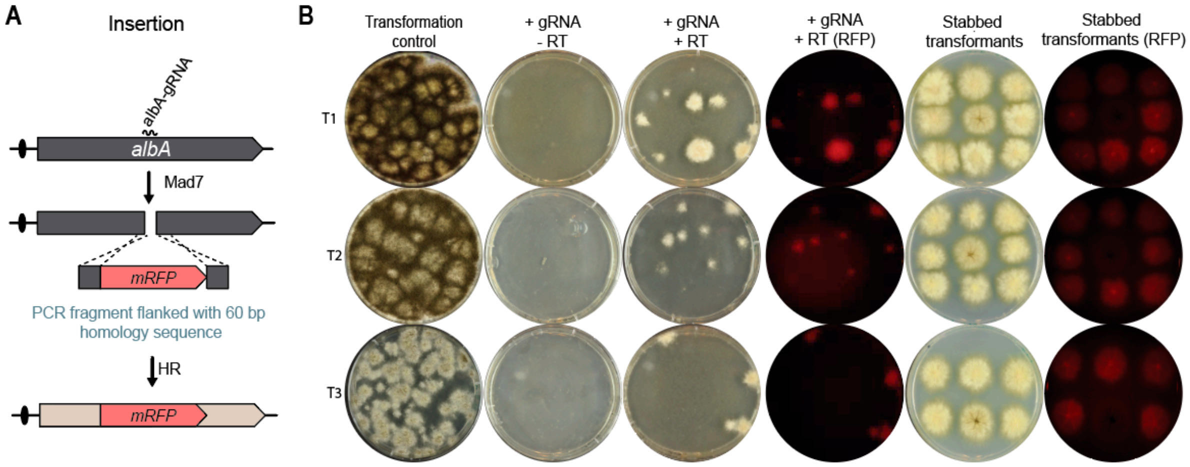

Supplementary Figure S5: Mad7 induced gene insertion in *A. niger*. A) Schematic representation of gene insertion experiments. Mad7 induces a DNA double-strand-break in the middle of a given gene. Subsequently, the DNA break is repaired by homologous recombination using a PCR fragment as repair template. In this case, the PCR fragment contains the RFP gene flanked by 60 bp of sequences identical to the two sides of the DNA break. B) Gene insertion of *albA* in *A. niger*. Transformation results for all trials. Transformation control are protoplasts transformed with the empty Mad7-CRISPR plasmids pDIV300. Transformation results of *A. niger* protoplasts transformed with *albA*-Mad7 CRISPR vectors in the presence and absence of an RFP PCR fragment, which is used as repair template as indicated. Stabs of independent transformants on a new plate are shown to the right with transformation control in the center of the plates
